# Prognostic Impact of Baseline Circulating Tumor DNA (ctDNA) in Pancreatic Ductal Adenocarcinoma: A Systematic Review and Meta-Analysis

**DOI:** 10.3390/cancers18142286

**Published:** 2026-07-16

**Authors:** Maen Abdelrahim, Abdullah Esmail, Minhal Zaidi, Nour Mustafa, Yazan Hamdaneh, Zaid Alabed, Saifudeen Abdelrahim, Ebtesam Al-Najjar, Hikmat Abdel-Razeq, Asem Mansour

**Affiliations:** 1 Section of GI Oncology, Department of Medicine, Houston Methodist Cancer Center, Houston, TX 77030, USA; aesmail@houstonmethodist.org (A.E.);; 2 Department of Medicine, Weill Cornell Medical College, New York, NY 10065, USA; 3 Faculty of Medicine, The University of Jordan, Amman 11942, Jordan; 4 Department of Internal Medicine, King Hussein Cancer Center, Amman 11941, Jordan; 5 Department of Radiology, King Hussein Cancer Center, Amman 11941, Jordan

**Keywords:** pancreatic ductal adenocarcinoma, circulating tumor DNA, overall survival, progression-free survival, meta-analysis

## Abstract

**Background**: Pancreatic ductal adenocarcinoma (PDAC) carries a dismal prognosis, with a 5-year overall survival of only 11% across all stages. Serum CA 19-9, the standard clinical biomarker, is limited by inadequate sensitivity in early stage disease and poor specificity. Circulating tumor DNA (ctDNA), which reflects the real-time genomic landscape of the tumor and frequently detects KRAS alterations present in over 90% of PDAC cases, has emerged as a promising prognostic biomarker. However, prior meta-analyses have been limited by smaller cohort sizes and substantial heterogeneity. This systematic review and meta-analysis aimed to evaluate the prognostic impact of detectable baseline ctDNA status in PDAC, irrespective of disease stage. **Methods**: A systematic search of Scopus, PubMed, and Embase was conducted for studies published up to January 2026, following PRISMA guidelines. Studies were eligible if they enrolled adults with PDAC, assessed ctDNA status (positive vs. negative) at baseline, and reported overall survival (OS) or progression-free survival (PFS). Case reports, reviews, studies focusing on specific mutations, or studies without extractable survival data were excluded. Hazard ratios (HRs) with 95% confidence intervals (CIs) were pooled using a random-effects model (DerSimonian–Laird method). Heterogeneity was assessed using the I^2^ statistic and Cochran’s Q test. Subgroup analyses were performed by disease stage (localized vs. advanced PDAC). **Results**: 14 studies encompassing 1032 patients were included; 487 (47.2%) had detectable baseline ctDNA. Overall, 482 patients (46.7%) had localized disease and 550 (53.3%) had advanced disease. Detectable baseline ctDNA was significantly associated with inferior OS across all 14 studies (pooled HR 2.33, 95% CI 1.98–2.75; *p* < 0.0001; I^2^ = 7.9%) and with shorter PFS across eight studies (pooled HR 2.16, 95% CI 1.76–2.67; *p* < 0.0001; I^2^ = 15.7%). In stage-stratified subgroup analyses, ctDNA positivity was independently associated with worse OS in both advanced PDAC (HR 2.21, 95% CI 1.77–2.75; I^2^ = 5%) and localized PDAC (HR 2.54, 95% CI 1.97–3.28; I^2^ = 14.6%), with no statistically significant difference between subgroups (*p* = 0.412). Similarly, ctDNA positivity predicted worse PFS in advanced (HR 2.19, 95% CI 1.69–2.82; I^2^ = 35.4%) and localized disease (HR 2.15, 95% CI 1.40–3.31; I^2^ = 0%), with no significant between-subgroup differences (*p* = 0.941). Heterogeneity was consistently low to moderate across analyses. **Conclusions**: Detectable baseline ctDNA is a potent, independent prognostic biomarker in PDAC, associated with more than a two-fold increased risk of death and disease progression irrespective of disease stage, with consistent findings across studies and ctDNA detection platforms. These findings support further prospective evaluation of baseline ctDNA for prognostic stratification, treatment personalization, and clinical trial enrichment before routine clinical implementation. Standardized assay methodologies and prospective validation are needed to facilitate its future integration into clinical practice.

## Simple Summary

Pancreatic cancer remains exceptionally difficult to treat, partly because current blood tracking markers fail to reliably capture hidden tumor spread or predict patient survival. This study aims to clarify whether measuring tumor genetic material shed into the bloodstream, known as circulating tumor DNA, before starting treatment can reliably identify high-risk patients. By pooling data from multiple studies, we demonstrate that detecting this genetic material indicates a significantly higher risk of rapid disease progression and shorter survival times, regardless of whether the cancer is early stage or advanced. These findings will contribute to the medical community by providing researchers and clinicians with a validated, minimally invasive tool to better categorize patient risk, customize aggressive frontline therapies, and design smarter clinical trials to improve overall survival outcomes.

## 1. Introduction

Pancreatic ductal adenocarcinoma is an aggressive malignancy that accounts for more than 90% of pancreatic cancers and remains one of the leading causes of cancer-related mortality worldwide [1,2,3]. Despite advances in diagnostic modalities and systemic therapies, PDAC continues to carry a dismal prognosis because of its aggressive tumor biology, late clinical presentation, and early metastatic spread [1,2,3]. More than half of patients present with metastatic disease at the time of diagnosis, limiting the potential for curative surgical intervention. Consequently, the overall 5-year survival rate remains at approximately 10%, with even poorer outcomes in advanced-stage disease. In addition, the incidence and mortality rates of PDAC continue to rise globally, and epidemiological projections suggest that it may become the second leading cause of cancer-related death by 2030 [1,2,3]. These poor outcomes highlight the urgent need for improved therapeutic strategies and a better understanding of tumor biology and treatment resistance in PDAC [4,5,6,7].

The aggressiveness and resistance of PDAC are driven by its unique tumor microenvironment (TME) that is characterized by a dense, fibrous capsule that acts as a physical barrier against immune cell infiltration and drug penetration, leading to limited efficacy of treatments like immune checkpoint inhibitors (ICIs) [2,8,9,10]. A key feature of PDAC’s molecular landscape is the high prevalence of mutations, including KRAS (in over 90% of cases), TP53, CDKN2A, SMAD4, BRCA1/2, and STK11; therefore, understanding these genomic features is critical for improving patient outcomes [2,8,9,10,11,12].

Currently, serum carbohydrate antigen 19-9 (CA 19-9) is the key biomarker widely used in PDAC management. However, its clinical utility is compromised by insufficient sensitivity, especially in early stage disease, and a lack of specificity due to elevated levels in benign conditions like pancreatitis and renal failure, or results in Lewis antigen-negative patients. This underscores an urgent need for a more precise, sensitive, and evidence-based prognostic and diagnostic biomarker [13,14,15].

PDAC is particularly well suited for ctDNA-based analysis because its molecular landscape is dominated by recurrent driver mutations, most notably KRAS, which is present in more than 90% of tumors, providing a stable genomic target for plasma-based detection [2,10]. Furthermore, the aggressive biology of PDAC promotes the continuous release of tumor-derived DNA into the circulation through tumor cell apoptosis and necrosis, allowing ctDNA to reflect tumor burden, occult metastatic disease, and disease dynamics in real time. These biological characteristics make ctDNA an attractive biomarker for prognostic assessment and longitudinal disease monitoring in PDAC [16,17,18].

Liquid biopsy has emerged as a promising non-invasive modality in oncology. Circulating tumor DNA (ctDNA) refers to tumor-derived DNA fragments released into the bloodstream, which harbor accurate tumor-specific somatic mutations. Assessment of ctDNA, which is amenable to serial sampling and reflects the real-time genomic features of the tumor, can potentially predict tumor volume, minimal residual disease (MRD), treatment response, and prognosis [16,19,20]. While prior studies and meta-analyses have explored the prognostic value of ctDNA in PDAC, substantial heterogeneity and smaller cohort sizes have limited the robustness of the pooled estimates. Therefore, in this systematic review and meta-analysis, we aim to provide robust, comprehensive evidence evaluating the prognostic impact of detectable baseline ctDNA status as an independent and stage-agnostic biomarker in patients with resectable or advanced pancreatic cancer.

## 2. Methodology

### 2.1. Search Strategy and Study Selection

**Figure 1:**
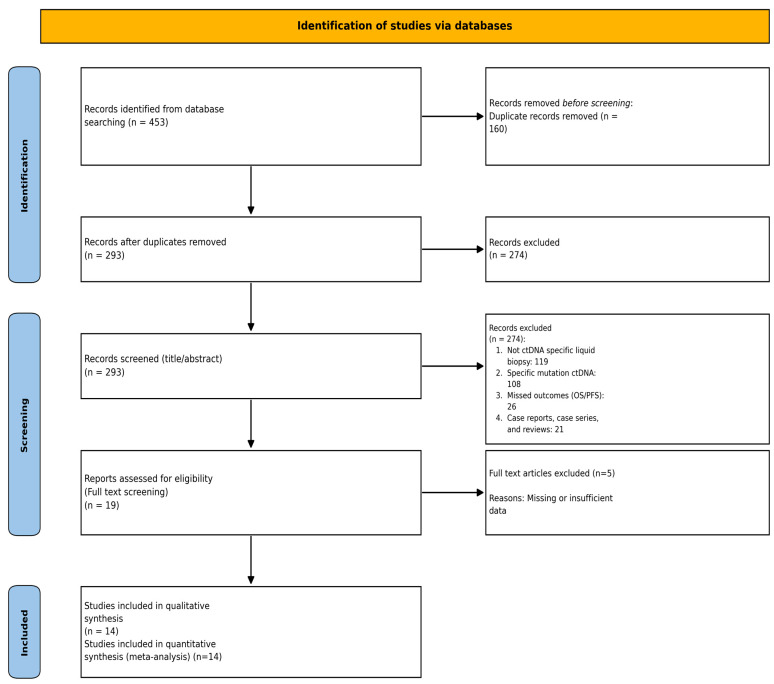
Flowchart of the literature search and study selection.

A systematic review of the medical literature was conducted in accordance with the Preferred Reporting Items for Systematic Reviews and Meta-Analyses (PRISMA 2020) guidelines. Although this review was not prospectively registered in PROSPERO, all review methods, including the eligibility criteria, search strategy, study selection, data extraction, and statistical analyses, were predefined before the review was conducted. The PRISMA 2020 checklist is provided in the Supplementary Materials. The PRISMA checklist can be found in Supplementary File S1. Investigators systematically searched the medical literature for any primary study that contained information about the prognostic role of ctDNA in pancreatic cancer. Electronic databases Scopus, PubMed, and Embase were queried for studies that fit our inclusion criteria. Studies published up to January 2026 were included in this meta-analysis. The electronic search criteria included the following keywords: (ctDNA OR circulating tumor DNA) AND (pancreatic cancer OR pancreatic carcinoma OR PDAC OR Pancreatic ductal adenocarcinoma) AND (prognosis OR prognostic impact OR outcome OR progression OR progress). A detailed search strategy per database is provided in Supplementary Material File S2. A summary of the search results and the PRISMA outline is shown in Figure 1.

Clinical trials and cohort studies are included if they focus on adults (more than 18 years old), studied pancreatic cancer or pancreatic ductal adenocarcinoma (PDAC), investigated baseline ctDNA status (positive or negative), and evaluated the overall survival or progression-free survival per baseline ctDNA status. Studies were excluded if they were case reports, case series, reviews, pooled analyses, commentaries, animal studies, papers with missing data, studies of a specific mutation in the DNA (specifically, those isolating a single rare or highly selected gene mutation rather than evaluating broader, clinically representative ctDNA panels or highly prevalent alterations such as KRAS panel shedding dynamics), studies focused on children (below 18 years), or studies of other types of cancer with ctDNA status. Literature selection was independently performed by two reviewers.

### 2.2. Data Extraction and Risk of Bias

Two investigators independently reviewed the titles and abstracts of all identified studies. Eligible studies underwent two rounds of independent screening consisting of title and abstract screening followed by full-text review. Disagreements regarding study inclusion were resolved by a third independent reviewer. The same two reviewers independently extracted the following data from the included studies: study identification (journal name, publication date, and ethics approval status), patient characteristics (sample size, treatment regimen, age, and gender), ctDNA status (positive or negative), and outcomes (hazard ratios for overall survival as the primary endpoint and progression-free survival). In this review, localized pancreatic cancer was defined as nonmetastatic disease without distant spread, whereas advanced cancer was considered to be locally advanced unresectable or metastatic disease based on the included studies.

Two investigators independently assessed the risk of bias of included studies using the Cochrane Risk of Bias in Non-Randomized Studies of Interventions (ROBINS-I) tool [21]. Risk of bias assessments were visualized using the robvis web application. All data were cleaned and analyzed using R software. Figures and tables were generated in R. The results were interpreted and written by the research team, with final manuscript editing and review being performed by senior authors.

### 2.3. Risk of Bias Tool Justification

The ROBINS-I tool was utilized for quality assessment because a substantial portion of the included literature evaluated patient cohorts undergoing specific, non-randomized therapeutic pathways (such as surgical resection versus systemic therapies), where inter-patient confounding variables closely mirror non-randomized intervention dynamics. While domain-specific tools like QUIPS (Quality In Prognostic Studies) are explicitly designed for prognostic factors, ROBINS-I was deemed appropriate to capture the intervention-dependent confounding inherent in these cohorts.

### 2.4. Cohort Overlap Assessment

A strict methodological review of studies originating from the same research groups was performed. No direct patient population or database overlap was identified, as subsequent publications functioned as post hoc analyses derived from entirely distinct clinical trial populations.

### 2.5. Statistical Analysis

The primary outcomes for this study were overall survival and progression-free survival, calculated from extracted univariate Cox proportional hazard regression models for overall survival (OS) and progression-free survival (PFS) in the included studies.

The analysis was conducted using R software version 4.4.2 (R Foundation for Statistical Computing). For prognostic outcomes, hazard ratios (HRs) with corresponding 95% confidence intervals (CIs) were pooled using a random-effects model (DerSimonian–Laird method), due to anticipated clinical and methodological heterogeneity among studies. The R packages used in this study are listed in Supplementary Material.

Statistical heterogeneity across studies was assessed using the I^2^ statistic and Cochran’s Q test. An I^2^ value of <25% was considered low heterogeneity, 25–50% was considered moderate, and >50% was considered high heterogeneity. In cases of substantial heterogeneity, subgroup and sensitivity analyses were performed to explore potential sources. Moreover, publication bias was assessed using a funnel plot that is shown in Figure S1.

## 3. Results

### 3.1. Study Characteristics

Out of 453 articles, 14 studies were included in the analysis. The characteristics of the included studies are shown in Table 1. The included studies were conducted across multiple geographic regions, with most of them conducted in the United States. Risk of bias analysis showed that eight studies had moderate risk while six studies had serious risk of bias, as shown in Figure S2.

A total of 1032 patients were included in the analysis; 487 (47.2%) had positive ctDNA prior to treatment. In total, 482 (46.7%) patients had localized disease while 550 (53.3%) patients had advanced disease, of them 176 and 311 had positive baseline ctDNA for localized and advanced PDAC, respectively. Mean ages ranged from 63 to 71 years, while the proportion of males ranged from 51.4% to 73.5%.

### 3.2. Survival Outcomes of All PDAC Patients

**Figure 2:**
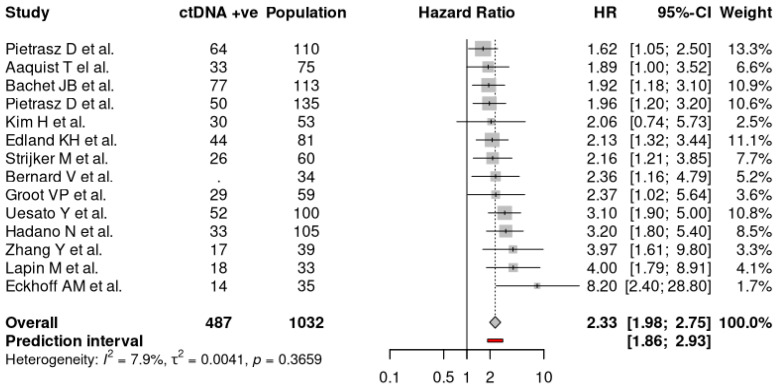
Forest plot of overall survival according to ctDNA status in pancreatic cancer with pooled random effects (HR OS of 2.33, 95% CI 1.98–2.75, *p* < 0.001, I^2^ = 7.9%) (all studies).

**Figure 3:**
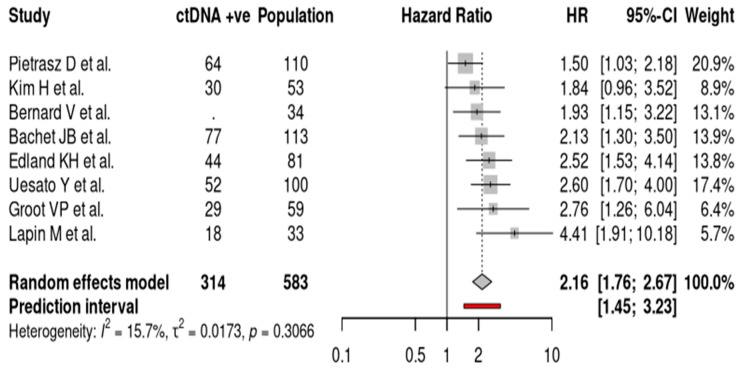
Forest plot of progression-free survival according to ctDNA status in pancreatic cancer with pooled random effects (HR PFS = 2.16, 95% CI 1.76–2.67, *p* < 0.001, I^2^ = 15.7%).

Across all studies, patients with positive baseline ctDNA had worse overall survival and progression-free survival compared to patients with negative baseline ctDNA, with pooled random effects for overall survival (HR OS of 2.33, 95% CI 1.98–2.75, *p* < 0.001, I^2^ = 7.9%), and pooled random effects for progression-free survival (HR PFS = 2.16, 95% CI 1.76–2.67, *p* < 0.001, I^2^ = 15.7%). Both analyses showed low heterogeneity between included studies, as shown in Figure 2 and Figure 3. Sensitivity analysis excluding the serious risk of bias studies did not change the results with the pooled HR for both OS (HR 2.06, 95% CI 1.70–2.49) and PFS (HR 1.96, 95% CI 1.57–2.44), as shown in the Supplementary Materials.

### 3.3. Differences in Survival Outcomes Between Localized and Advanced PDAC Patients

**Figure 4:**
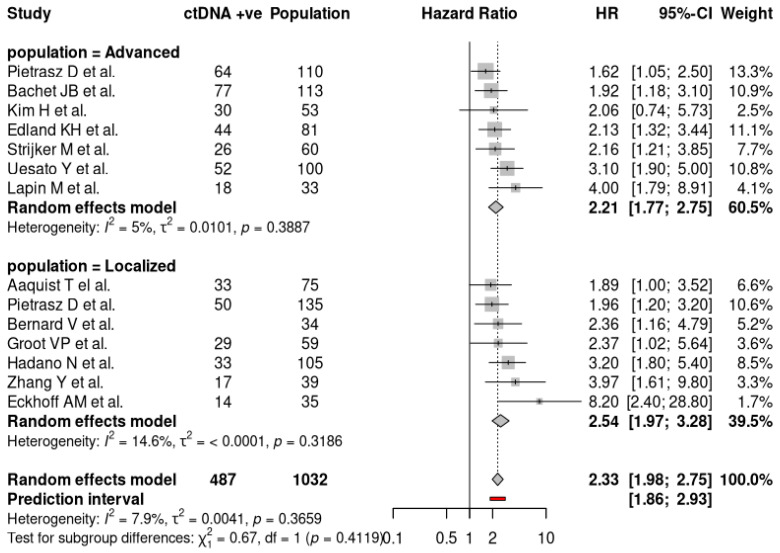
Forest plot of overall survival according to ctDNA in localized (resectable) vs. advanced pancreatic cancer, with pooled hazard ratios of 2.21 (95% CI 1.77–2.75, *p* < 0.001, I^2^ = 5%) for advanced disease and 2.54 (95% CI 1.97–3.28, *p* < 0.001, I^2^ = 14.6%) for localized disease.

As shown in Figure 4, positive baseline ctDNA was also associated with worse overall survival in patients with advanced and localized PDAC (HR = 2.21, 95% CI 1.77–2.75, *p* < 0.001, I^2^ = 5%) and (HR = 2.54 95% CI 1.97–3.28, *p* < 0.001, I^2^ = 14.6%), respectively, and there was no significant difference in the HR between both groups, with a *p*-value of 0.412. This subgroup analysis should be interpreted cautiously, as the analysis may have been underpowered to detect differences between disease stages.

**Figure 5:**
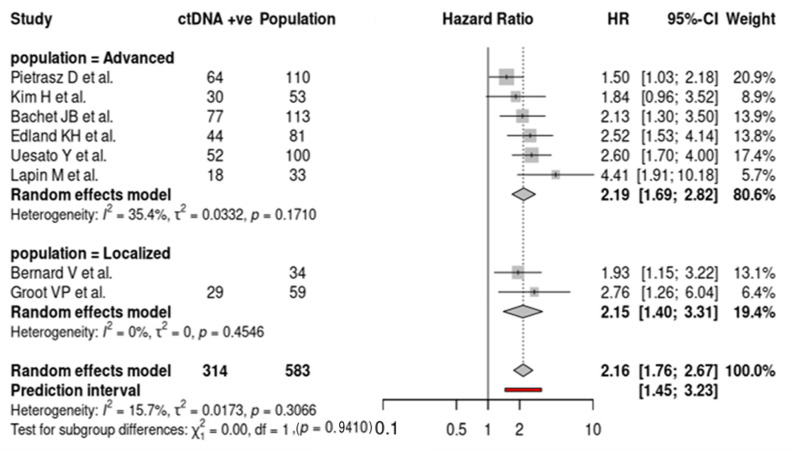
Forest plot of progression-free survival according to ctDNA status in localized (resectable) vs. advanced pancreatic cancer, with pooled hazard ratios of 2.19 (95% CI 1.69–2.82, *p* < 0.001, I^2^ = 35.4%) for advanced disease and 2.15 (95% CI 1.4–3.31, *p* < 0.001, I^2^ = 0%) for localized disease.

Additionally, positive baseline ctDNA was associated with worse prognosis in patients with advanced and localized PDAC. The pooled hazard ratios for advanced and localized PDAC patients were (HR = 2.19, 95% CI 1.69–2.82, *p* < 0.001, I^2^ = 35.4%) and (HR = 2.15, 95% CI 1.4–3.31, *p* < 0.001, I^2^ = 0%), respectively. No significant difference between both subgroups was found, with a *p*-value of 0.941. There was low heterogeneity between the studies included in Figure 5. The I^2^ values were low for localized PFS, where only two studies contributed to the analysis, as a small number of studies can limit the ability to detect true variability. Table 2 shows the summarized pooled hazard ratios for OS and PFS.

## 4. Discussion

In this study, we synthesized data from 14 studies encompassing 1032 patients with PDAC to evaluate the prognostic significance of baseline pre-treatment ctDNA status. Our principal finding is that detectable baseline ctDNA was consistently associated with significantly inferior survival outcomes irrespective of disease stage. Specifically, patients with detectable baseline ctDNA experienced markedly worse overall survival (pooled HR 2.33, 95% CI 1.98–2.75) and progression-free survival (pooled HR 2.16, 95% CI 1.76–2.67) compared with patients with undetectable ctDNA. These findings were remarkably consistent throughout the studies, with low statistical heterogeneity throughout the analyses.

### 4.1. Prognostic Landscape of PDAC and the Rationale for Liquid Biopsy

Given the aggressive biology and poor prognosis of pancreatic ductal adenocarcinoma, there is a critical need for reliable prognostic biomarkers capable of improving risk stratification and treatment personalization [35,36]. Although CA 19-9 remains the most widely used biomarker in PDAC, its clinical utility is limited by suboptimal sensitivity and specificity, particularly in early stage disease and in Lewis antigen-negative patients. In this context, ctDNA has emerged as a promising non-invasive biomarker that may better reflect real-time tumor burden and disease biology [15].

Liquid biopsy, defined as the non-invasive sampling and analysis of tumor-derived fragments in peripheral blood, has emerged as a solid and accurate approach in oncology. ctDNA, which represents tumor-derived cell-free DNA fragments harboring somatic mutations characteristic of the underlying malignancy, may reflect the real-time genomic features of the tumor [17,18]. Unlike tissue biopsy, ctDNA assessment circumvents issues of spatial tumor heterogeneity, is amenable to serial sampling, and can be performed with minimal patient discomfort. In PDAC, the high prevalence of targetable KRAS mutations which are detected in over 90% of cases provides an ideal substrate for mutation-specific ctDNA detection, making PDAC particularly well suited for ctDNA-based liquid biopsy approaches [37].

### 4.2. Baseline ctDNA and Overall Survival

The pooled analysis of all 14 studies included in our work demonstrated a significant association between detectable baseline ctDNA and inferior OS (HR 2.33, 95% CI 1.98–2.75; I^2^ = 7.9%). This association is biologically plausible, as detectable ctDNA likely reflects higher tumor burden, increased tumor cell turnover, and occult micrometastatic disease, all of which are associated with poorer survival outcomes. Detectable ctDNA may also identify tumors with more aggressive biological behavior and greater metastatic potential. Importantly, the I^2^ statistic of 7.9% and the non-significant Q-test (*p* = 0.37) indicate near-absent heterogeneity, suggesting that the prognostic value of baseline ctDNA for OS is consistent across different PDAC populations, treatment regimens, and ctDNA detection methods included in this analysis. This finding is concordant with prior individual studies and an earlier meta-analysis by Fang et al., which reported a pooled HR for the OS of approximately 2.1 in ctDNA-positive PDAC patients, though that analysis may have been limited by potentially high heterogeneity [38].

### 4.3. Baseline ctDNA and Progression-Free Survival

Analysis of PFS across eight studies demonstrated that ctDNA positivity was associated with a significantly shorter time to disease progression or death (pooled HR 2.16, 95% CI 1.76–2.67; I^2^ = 15.7%). This finding is consistent with the biological premise that tumors shedding detectable ctDNA are generally more aggressive and more likely to progress earlier than tumors with undetectable ctDNA.

The consistency of this association suggests that early tumor dissemination detectable via baseline ctDNA reflects cancer aggressiveness that translates uniformly into accelerated disease progression. This is also biologically plausible as higher ctDNA levels at baseline are likely a surrogate of greater tumor burden, higher cell turnover, and more active DNA shedding, all of which translate into aggressive disease kinetics and early metastatic dissemination [39,40].

### 4.4. Subgroup Analysis by Disease Stage

In the OS analysis of all 14 studies, detectable ctDNA was associated with inferior survival in both the advanced disease subgroup (HR 2.21, 95% CI 1.77–2.75; I^2^ = 5%, comprising seven studies) and the localized disease subgroup (HR 2.54, 95% CI 1.97–3.28; I^2^ = 14.6%, comprising eight studies). The test for subgroup differences was non-significant (*p* = 0.412), indicating no statistically significant difference in the magnitude of the ctDNA prognostic effect between disease stages.

These results are of high clinical significance. While it might be expected that ctDNA detection in advanced disease reflects established metastatic burden, the demonstration of an equally strong prognostic association in resectable or localized PDAC is of paramount importance. In this latter population, where surgical resection is pursued with curative intent, a positive baseline ctDNA may reflect occult micrometastatic disease not detectable by conventional means [30].

### 4.5. Biological Underpinnings of ctDNA as a Prognostic Marker in PDAC

The consistent prognostic value of ctDNA across PDAC stages and treatment settings likely reflects multiple intersecting biological mechanisms. First of all, ctDNA levels at baseline serve as a proxy for total tumor burden and tumor shedding rate, both of which are direct determinants of survival in solid tumors [24,41]. Secondly, KRAS-mutant ctDNA, which represents the most commonly targeted analyte in PDAC liquid biopsy studies given the ubiquitous presence of KRAS mutations in PDAC, reflects the clonal dominance of transformed epithelium and has been shown to correlate with tumor burden and dissemination [38].

Moreover, in the context of ctDNA shed from micrometastatic deposits, as may be the case in apparently localized PDAC, ctDNA positivity may reflect viable, proliferating cancer cells at distant sites that are below the spatial resolution of current imaging modalities.

### 4.6. Comparison with Prior Systematic Reviews and Meta-Analyses

Several prior meta-analyses have examined the prognostic value of ctDNA in PDAC, although the field has evolved rapidly as new studies have been published. Fang et al. reported pooled OS and PFS multivariate HRs of 2.07 (95% CI 1.64–2.58) and 2.2 (95% CI 1.82–3.19), respectively, in an earlier meta-analysis, findings directionally consistent with our results [38]. However, the authors of that paper reported possible high heterogeneity, potentially limiting confidence in the pooled estimates.

Similarly, a meta-analysis by Chen et al. demonstrated that ctDNA positivity was significantly associated with worse OS (multivariate HR = 2.57, 95% CI: 1.95–3.38, I^2^ = 66%) and PFS (multivariate HR = 2.31, 95% CI: 1.47–3.64, I^2^ = 0%), although the high I^2^ statistic value may signal high heterogeneity risk [42].

The current meta-analysis extends this body of evidence by including a larger and more contemporary cohort of studies (14 studies for OS and eight for PFS) and providing stage-stratified subgroup analyses. Importantly, despite differences in study populations, disease stage, and ctDNA detection methods, our pooled hazard ratios remained highly consistent with those reported in previous meta-analyses. This consistency suggests that the prognostic impact of baseline ctDNA is robust across diverse clinical settings. Furthermore, the lower heterogeneity observed in our analysis may reflect improved methodological consistency among more recent studies. The separate sensitivity analysis restricted to the PFS study subset further strengthens the internal validity of our findings.

### 4.7. Clinical Implications and Translational Relevance

The findings of this comprehensive meta-analysis suggest several potential clinical implications that warrant further prospective evaluation for the management of PDAC. To begin with, baseline ctDNA testing at the time of diagnosis may serve as a valuable biomarker for refined prognostic stratification. The consistently strong association between detectable ctDNA and inferior survival, even in localized disease, suggests that ctDNA positivity may reflect occult micrometastatic disease not detectable by conventional imaging. This high-risk subset of resectable patients may represent candidates for evaluation in future prospective studies investigating intensified treatment strategies. Additionally, ctDNA quantification and status at baseline can serve as a crucial patient-selection and enrichment tool in clinical trials of novel therapeutics. Beyond simply selecting patients for targeted agents, such as KRAS inhibitors, ctDNA positivity can be used as a stratification variable in randomized trials to ensure comparable high-risk cohorts, particularly when evaluating novel agents like combination immunotherapy strategies.

Moreover, the ability of ctDNA to reflect real-time tumor dynamics facilitates its use in serial monitoring. Serial ctDNA has the potential to guide future treatment decisions, although prospective validation is required before its routine clinical implementation. This can potentially guide timely intervention, such as dose intensification or treatment switch, to mitigate disease recurrence.

This study must be interpreted in the context of its limitations. Despite a comprehensive literature search, the majority of included studies were retrospective in design, with inherent risks of confounding and selection bias. Prospective, protocol-driven ctDNA assessments integrated within randomized trials would provide higher-level evidence. Potential confounders include differences in disease stage, treatment regimens, patient selection, and baseline clinical characteristics, all of which may have influenced the observed association between ctDNA positivity and survival.

In addition, considerable inter-study heterogeneity exists in ctDNA detection methodology, mutation targets interrogated, ctDNA positivity thresholds, and timing of blood sampling relative to treatment initiation. While the low I^2^ statistics in our pooled analyses provide some reassurance, residual heterogeneity at the methodological level might not be fully captured by the I^2^ metric, particularly given the relatively small number of studies in certain subgroups. Most included studies used PCR-based assays targeting KRAS mutations, whereas others used NGS-based platforms. Differences in assay sensitivity, target selection, and detection thresholds may have contributed to variability across studies.

Moreover, the subgroup analysis by disease stage was limited by the small number of studies with localized/resectable PDAC (two studies for OS in the full cohort and seven for advanced disease), which constrains the precision of the localized disease estimates and reduces statistical power for subgroup comparisons. The wide confidence intervals observed in the localized disease OS subgroup (HR 2.54, 95% CI 1.97–3.28) reflect this limitation.

Furthermore, ctDNA has inherent limitations as a biomarker, including the potential for false-negative results in tumors with low DNA shedding and variability in assay performance across different detection platforms. These factors should be considered when interpreting ctDNA results. Furthermore, the absence of standardized ctDNA positivity thresholds across studies may have affected patient classification and comparability, highlighting the need for harmonized assay protocols and reporting standards in future prospective studies.

### 4.8. Future Directions

Several important research priorities should emerge from this study. Prospective, multi-center studies integrating standardized ctDNA assessments with pre-specified assay platforms, mutation panels, positivity thresholds, and sampling time points are needed to generate practice-changing evidence.

In addition, future ctDNA-stratified randomized phase II clinical trials, particularly in patients with resectable PDAC, should evaluate whether baseline ctDNA status can guide treatment intensification or patient selection for neoadjuvant and adjuvant therapies. Comparative studies evaluating KRAS mutation-specific assays alongside tumor-agnostic methylation-based approaches, such as those described by Lapin et al., are also needed to determine the optimal platform for standardization and routine clinical implementation.

Future work should also examine the incremental prognostic value of ctDNA over and above CA 19-9 and other established biomarkers. The combination of ctDNA with other liquid biopsy analytes, such as circulating tumor cells, exosomal nucleic acids, and methylation-based cell-free DNA profiling, may yield superior prognostic composites. Additionally, the investigation of ctDNA dynamics as a pharmacodynamic biomarker and its utility in guiding treatment de-escalation or intensification decisions in real time may represent a promising approach that needs to be further validated by future studies.

## 5. Conclusions

This comprehensive systematic review and meta-analysis, encompassing 1032 PDAC patients across 14 studies, provides robust, comprehensive evidence that detectable baseline ctDNA is a potent prognostic biomarker in PDAC irrespective of disease stage. Patients with detectable pre-treatment ctDNA face an increased risk of death and disease progression, with this association remaining consistent across resectable and advanced disease stages and across diverse ctDNA detection methods, as evidenced by remarkably low statistical heterogeneity. These findings support further prospective evaluation of baseline ctDNA as a prognostic biomarker. If validated in prospective studies using standardized assays, baseline ctDNA may be incorporated into future PDAC management algorithms for risk stratification and clinical trial enrichment.

## Figures and Tables

**Table 1:** Characteristics of included studies.

Study Name	Year	Country	Intervention	Sample Type	Stage	+ctDNA/Sample Size	Reference
Kim H et al.,	2025	Korea	ctDNA	Blood	Advanced	30/53	[22]
Pietrasz D et al.	2021	France	ctDNA	Blood	Advanced	64/110	[23]
Edland KH et al.	2023	Norway	ctDNA	Blood	Advanced	44/81	[19]
Strijker M et al.	2020	The Netherland, Italy	ctDNA	Blood	Advanced	26/60	[24]
Uesato Y et al.	2020	Japan	ctDNA	Blood	Advanced	52/100	[25]
Pietrasz D et al.	2017	France	ctDNA	Blood	Advanced	50/135	[26]
Bernard V et al.	2019	US	ctDNA	Blood	Localized	17/34	[27]
Bachet JB et al.	2020	France	ctDNA	Blood	Advanced	77/113	[28]
Hadano N et al.	2016	Japan	ctDNA	Blood	Localized	33/105	[29]
Groot VP et al.	2019	US	ctDNA	Blood	Localized	29/59	[30]
Zhang Y et al.	2025	US	ctDNA	Blood	Localized	17/39	[31]
Eckhoff AM et al.	2024	US	ctDNA	Blood	Localized	14/35	[32]
Aaquist T et al.	2025	Denmark, Sweden, Germany	ctDNA	Blood	Localized	33/75	[33]
Lapin M et al.	2025	Norway	ctDNA	Blood	Advanced	18/33	[34]

**Table 2:** Summary of pooled hazard ratios for overall survival and progression-free survival by disease stage.

Disease Group	OS Pooled HR (95% CI)	*p* Value	PFS Pooled HR (95% CI)	*p* Value
All studies	2.33 (1.98–2.75)	<0.001	2.16 (1.76–2.67)	<0.001
Localized PDAC	2.54 (1.97–3.28)	<0.001	2.15 (1.40–3.31)	<0.001
Advanced PDAC	2.21 (1.77–2.75)	<0.001	2.19 (1.69–2.82)	<0.001

## Data Availability

The data supporting the findings of this meta-analysis, entitled “Prognostic Impact of Baseline Circulating Tumor DNA (ctDNA) in Pancreatic Ductal Adenocarcinoma: A Systematic Review and Meta-Analysis”, are available upon request from the corresponding author, Maen Abdelrahim (mabdelrahim@houstonmethodist.org).

## Supplementary Materials

The following supporting information can be downloaded at: https://www.mdpi.com/article/10.3390/cancers18142286/s1, Figure S1: Funnel plot using 14 included studies about the prognostic role of pretreatment ctDNA; Figure S2: Risk of bias assessment graph for all included studies; Supplementary File S1: PRISMA-ScR-Fillable-Checklist. Supplementary File S2: Search Protocol.
