# Supplementary material for: Prognostic Impact of Baseline Circulating Tumor DNA (ctDNA) in Pancreatic Ductal Adenocarcinoma: A Systematic Review and Meta-Analysis
Source: Cancers (Basel). 2026 Jul 16;18(14):2286. doi: 10.3390/cancers18142286 (PMC13407315; doi:10.3390/cancers18142286)
Supplement: Supplementary file 1 [file cancers-18-02286-s001.zip › cancers-4394346 supp.pdf]

Supplemental 1

List of R packages used in the analysis and the manuscript:

readr

readxl

pacman

ggplot2

stringr

rio

tidyr

tidyverse

meta

metafor

PRISMA2020

robvis

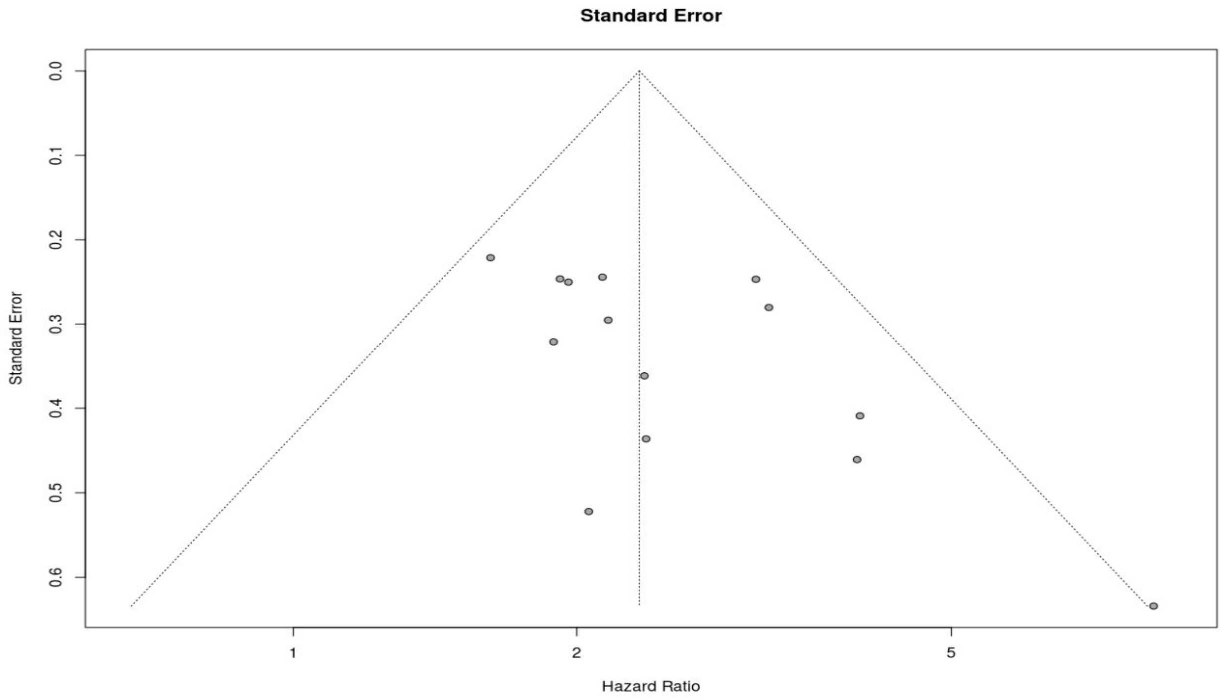

Figure S1: Funnel plot using 14 included studies about the prognostic role of pretreatment ctDNA.

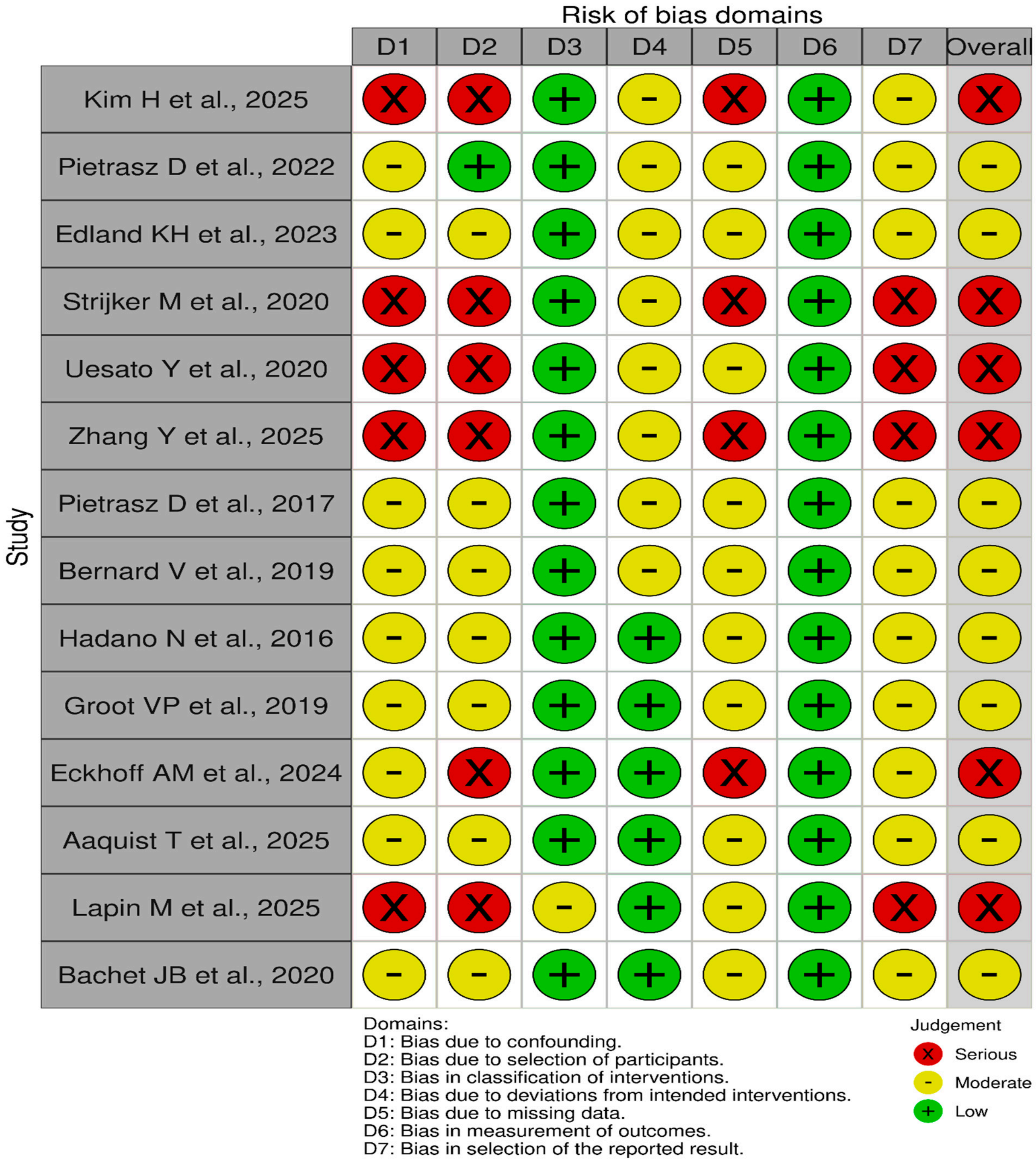

Figure S2: Risk of Bias assessment graph for all included studies
