# Supplementary material for: Prognostic Impact of Baseline Circulating Tumor DNA (ctDNA) in Pancreatic Ductal Adenocarcinoma: A Systematic Review and Meta-Analysis
Source: Cancers (Basel). 2026 Jul 16;18(14):2286. doi: 10.3390/cancers18142286 (PMC13407315; doi:10.3390/cancers18142286)
Supplement: Supplementary file 1 [file cancers-18-02286-s001.zip › File S2 Search Protocol .pdf]

Search number	Query	SEARCH DETAILS
3	# 1 AND # 2 AND # 3	(((((Liquid Biopsy[MeSH Terms]) OR (circulating tumor DNA[Title/Abstract])) OR (circulating tumour DNA[Title/Abstract] OR ctDNA[Title/Abstract])) OR (cell-free DNA[Title/Abstract])) OR (cell-free DNA[Title/Abstract] OR cell free DNA[Title/Abstract] OR cfDNA[Title/Abstract] OR liquid biopsy[Title/Abstract])) AND ((("Pancreatic Neoplasms"[Mesh] OR "pancreatic cancer"[Title/Abstract] OR "pancreatic carcinoma"[Title/Abstract] OR "pancreatic adenocarcinoma"[Title/Abstract] OR "pancreatic ductal adenocarcinoma"[Title/Abstract] OR PDAC[Title/Abstract] OR "pancreatic neoplasm"[Title/Abstract]))) AND ("Overall survival"[Title/Abstract] OR "progression-free survival"[Title/Abstract])
3	"Overall survival"[Title/Abstract] OR "progression-free survival"[Title/Abstract]	"Overall survival"[Title/Abstract] OR "progression-free survival"[Title/Abstract]
2	(("Pancreatic Neoplasms"[Mesh] OR "pancreatic cancer"[Title/Abstract] OR "pancreatic carcinoma"[Title/Abstract] OR "pancreatic adenocarcinoma"[Title/Abstract] OR "pancreatic ductal adenocarcinoma"[Title/Abstract] OR PDAC[Title/Abstract] OR "pancreatic neoplasm"[Title/Abstract]))	(("Pancreatic Neoplasms"[Mesh] OR "pancreatic cancer"[Title/Abstract] OR "pancreatic carcinoma"[Title/Abstract] OR "pancreatic adenocarcinoma"[Title/Abstract] OR "pancreatic ductal adenocarcinoma"[Title/Abstract] OR PDAC[Title/Abstract] OR "pancreatic neoplasm"[Title/Abstract]))
1	((((Liquid Biopsy[MeSH Terms]) OR (circulating tumor DNA[Title/Abstract])) OR (circulating tumour DNA[Title/Abstract] OR ctDNA[Title/Abstract])) OR (cell-free DNA[Title/Abstract])) OR (cell-free DNA[Title/Abstract] OR cell free DNA[Title/Abstract] OR cfDNA[Title/Abstract] OR liquid biopsy[Title/Abstract])	((((Liquid Biopsy[MeSH Terms]) OR (circulating tumor DNA[Title/Abstract])) OR (circulating tumour DNA[Title/Abstract] OR ctDNA[Title/Abstract])) OR (cell-free DNA[Title/Abstract])) OR (cell-free DNA[Title/Abstract] OR cell free DNA[Title/Abstract] OR cfDNA[Title/Abstract] OR liquid biopsy[Title/Abstract])

PUBMED SEARCH PROTOCOL

SCOPUS SEARCH PROTOCOL:

TITLE-ABS-KEY("circulating tumor DNA" OR "circulating tumour DNA" OR ctDNA OR "cell-free DNA" OR "cell free DNA" OR cfDNA OR "liquid biopsy") **AND** TITLE-ABS-KEY("pancreatic cancer" OR "pancreatic carcinoma" OR "pancreatic adenocarcinoma" OR "pancreatic ductal adenocarcinoma" OR PDAC OR "pancreatic neoplasm")

EMBASS search protocol

('liquid biopsy'/exp OR 'circulating tumor dna':ti,ab OR 'circulating tumour dna':ti,ab OR ctdna:ti,ab OR 'cell-free dna':ti,ab OR 'cell free dna':ti,ab OR cfdna:ti,ab) **AND** ('pancreas cancer'/exp OR 'pancreas tumor'/exp OR 'pancreatic cancer':ti,ab OR 'pancreatic carcinoma':ti,ab OR 'pancreatic adenocarcinoma':ti,ab OR 'pancreatic ductal adenocarcinoma':ti,ab OR PDAC:ti,ab OR 'pancreatic neoplasm':ti,ab)
